# Advanced Maternal Age Impairs Uterine Artery Adaptations to Pregnancy in Rats

**DOI:** 10.3390/ijms23169191

**Published:** 2022-08-16

**Authors:** Amy L. Wooldridge, Mazhar Pasha, Palehswan Chitrakar, Raven Kirschenman, Anita Quon, Floor Spaans, Tamara Sáez, Christy-Lynn M. Cooke, Sandra T. Davidge

**Affiliations:** 1 Department of Obstetrics and Gynecology and Women and Children’s Health Research Institute, University of Alberta, Edmonton, AB T6G 2R3, Canada; 2 Department of Physiology, University of Alberta, Edmonton, AB T6G 2R3, Canada

**Keywords:** advanced maternal age, uterine artery, pregnancy, myogenic response, circumferential stress-strain, arterial structure, collagen, elastin, myography

## Abstract

Advanced maternal age (≥35 years) is associated with pregnancy complications. Aging impairs vascular reactivity and increases vascular stiffness. We hypothesized that uterine artery adaptations to pregnancy are impaired with advanced age. Uterine arteries of nonpregnant and pregnant (gestational day 20) young (4 months) and aged (9 months; ~35 years in humans) Sprague-Dawley rats were isolated. Functional (myogenic tone, n = 6–10/group) and mechanical (circumferential stress-strain, n = 10–24/group) properties were assessed using pressure myography and further assessment of elastin and collagen (histology, n = 4–6/group), and matrix metalloproteinase-2 (MMP-2, zymography, n = 6/group). Aged dams had worse pregnancy outcomes, including smaller litters and fetal weights (both *p* < 0.0001). Only in arteries of pregnant young dams did higher pressures (>100 mmHg) cause forced vasodilation. Across the whole pressure range (4–160 mmHg), myogenic behavior was enhanced in aged vs. young pregnant dams (*p* = 0.0010). Circumferential stress and strain increased with pregnancy in young and aged dams (*p* < 0.0001), but strain remained lower in aged vs. young dams (*p* < 0.05). Arteries from young nonpregnant rats had greater collagen:elastin ratios than the other groups (*p* < 0.05). In aged rats only, pregnancy increased MMP-2 active capacity. Altered functional and structural vascular adaptations to pregnancy may impair fetal growth and development with advanced maternal age.

## 1. Introduction

A growing number of women have pregnancies at an advanced maternal age (35 years), such that 25% of all births in Canada now occur in women aged at least 35 years [1]. This is a growing and under-appreciated health problem since advanced maternal age is a well-known risk factor for developing life-threatening complications during pregnancy, such as preeclampsia and fetal growth restriction [2,3]. This increased risk for complications may be due to impaired pregnancy adaptations, and in particular, impaired uteroplacental vascular adaptations that occur during pregnancy [4].

A healthy pregnancy undergoes massive maternal hemodynamic adaptations, including an increase in blood volume, a 50% increase in cardiac output, and a decrease in peripheral vascular resistance [5]. Moreover, extensive transformation and remodeling of uteroplacental vasculature are critical to increasing artery diameter, facilitating sufficient blood supply to transport nutrients and oxygen to the placenta and developing fetus. Insufficient remodeling of the uteroplacental vasculature (i.e., impaired vascular adaptations to pregnancy) contributes to pregnancy complications such as preeclampsia, fetal hypoxia, and subsequent fetal growth restriction [6,7,8].

An important pregnancy-induced adaptation within the uteroplacental vasculature is altered myogenic tone, defined as the intrinsic property of the vascular smooth muscle to contract in response to changes in the outward stretch to maintain vessel diameter. The myogenic tone thus functions as an autoregulatory process to maintain constant blood flow to critical organs across varying blood pressures [9]. The myogenic tone was reported to be increased (human myometrial arteries and rat radial uterine arteries [10,11,12,13,14,15]) or decreased (mouse and guinea pig main uterine arteries [16,17,18]) with pregnancy. In addition to vascular function, the structure of arteries also determines their capacity for blood flow. Outward hypertrophic remodeling of the main uterine arteries during pregnancy is essential for enabling sufficient blood flow to the fetoplacental unit [19]. This remodeling results in greater passive lumen diameter and altered extracellular matrix arrangement [20,21]. Mechanical stress and strain properties of arteries are altered by structural proteins in the vascular wall, such as elastin and collagen, and the expression of these proteins is altered with pregnancy through degradation and turnover to facilitate remodeling [22,23,24]. Age-related changes are not uniform throughout the vasculature [25], and there is limited information on uterine arteries. A study of main uterine arteries from 40-week-old (aged) mice reported a lack of increased diameter with pregnancy compared with arteries from 12-week-old (young) mice [24]. This suggests that the factors responsible for structural remodeling may alter with advanced maternal age. However, it is unknown how aging may affect the pregnancy-related adaptations in myogenic tone and stress-strain properties of the main uterine artery specifically.

The degradation of structural proteins such as elastin and collagen in the vascular wall is regulated by matrix metalloproteinases (MMPs) such as MMP-2 and -9. MMPs are known to be altered with pregnancy (reviewed in [26]) and aging (reviewed in [27]). During normal pregnancies, MMP-2 and MMP-9 expressions are elevated in uterine arteries, contributing to the remodeling of these arteries [28]. Reduced levels of MMP-2 and -9 are implicated in pregnancy complications such as preeclampsia [29,30], but the extent to which these changes occur in advanced maternal age is unknown. MMP-2 and -9 positively correlate with circulating estrogen levels during pregnancy [31], and first-trimester circulating estrogen levels are negatively correlated with maternal age [32,33,34]. Estrogen can enhance the release of MMPs from vascular smooth muscle cells while promoting greater vasodilatory responses in uterine arteries [35,36,37], thus implicating estrogen as a potential age-sensitive mediator of uterine vascular function and remodeling.

Overall, advanced maternal age may result in poor pregnancy outcomes via abnormal uteroplacental vascular adaptations to pregnancy [4]. We have previously shown that uterine arteries from pregnant dams of advanced maternal age had greater active myogenic responses than arteries from young pregnant controls [38], which could be due to altered vascular adaptations in response to pregnancy. It is not known whether altered vascular function existed prior to pregnancy or whether it developed only during gestation as a result of altered pregnancy-induced remodeling. In the current study, we used a rat model for advanced maternal age to assess pregnancy-induced changes in uterine artery function and structure in young and aged rats compared to the nonpregnant state. We hypothesized that vascular adaptations in uterine arteries become impaired (i.e., less extensive) with advanced maternal age.

## 2. Results

### 2.1. Aged Dams Had Worse Pregnancy Outcomes Than Young Dams

**Figure 1:**
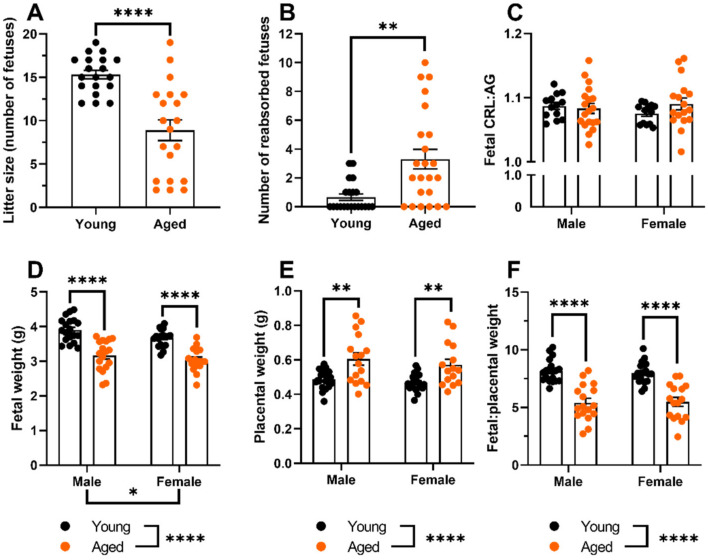
Pregnancy outcomes from young and aged dams at GD 20. (**A**) Litter size; (**B**) Reabsorptions; (**C**) Fetal crown-rump length (CRL):abdominal girth (AG) ratio; (**D**) Fetal weight; (**E**) Placental weight; (**F**) Fetal:placental weight ratio. Data presented as mean ± SEM; n represents litter average; n = 13–19 dams/group. Data are sex-specific means of litter outcomes, where applicable. Pregnancy outcomes were compared using a *t*-test (litter size), Mann Whitney U-test (reabsorptions), or a two-way ANOVA (dam age vs. fetal sex) followed by Sidak’s post hoc test. * *p* < 0.05; ** *p* < 0.01; **** *p* < 0.0001.

Aged dams had smaller litter sizes (*p* < 0.0001, Figure 1A) and more reabsorptions (*p* = 0.0014; Figure 1B) compared to the young dams. Fetuses from aged dams had reduced fetal weights (both males and females; *p* < 0.0001, Figure 1D) compared with young dams. The crown-rump length abdominal girth ratio was not different between fetuses from aged and young dams (Figure 1C). Fetuses from aged dams had a greater placental weight (males: *p* = 0.0013, females: *p* = 0.0059; Figure 1E) compared with young dams. The fetal placental weight ratio was lower in both fetal males and females of aged dams compared to young dams (both *p* < 0.0001, Figure 1F).

### 2.2. Myogenic Tone Was Reduced Only the Uterine Arteries of Young, but Not Aged, Pregnant Dams; Which Showed Forced Vasodilation at High Intraluminal Pressures

**Figure 2:**
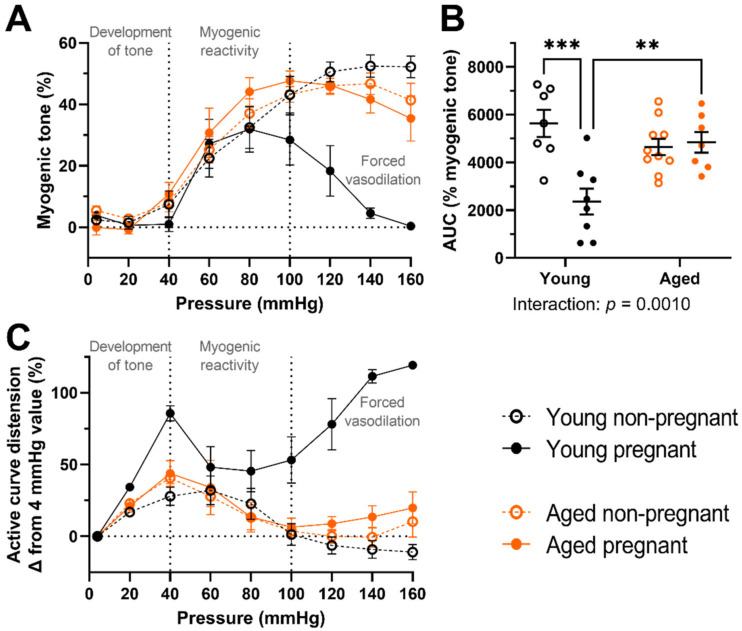
Active pressure myography outcomes for main uterine arteries from young (black) and aged (orange) nonpregnant (open circles) and pregnant (GD 20; closed circles) rats. (**A**) Percentage myogenic tone calculated from the intraluminal diameter (ID) measures using the equation: percentage myogenic tone = ((ID_passive_ − ID_active_)/ID_passive_) × 100, where ID_active_ is the intraluminal diameter when assessed in phosphate-buffered saline solution with calcium present to enable smooth muscle cell contraction, and ID_passive_ is the intraluminal diameter when assessed in a calcium-free solution containing papaverine to prevent smooth muscle contraction; (**B**) Percentage myogenic tone area under the curve (AUC), calculated from the entire pressure curve range of 4–160 mmHg; (**C**) The active pressure myography curve data, now presented as a percentage difference in ID from baseline (4 mmHg). Data presented as mean ± SEM; n = 7–10 rats/group. Percentage myogenic tone AUC was compared using a two-way ANOVA (age vs. pregnancy status) followed by Sidak’s post hoc test. ** *p* < 0.01; *** *p* < 0.001.

Myogenic tone decreased with pregnancy in the main uterine arteries of young rats (*p* = 0.0029, Figure 2A–C), which did not occur in the aged rats. Notably, at intraluminal pressures of 100 mmHg and above, there was forced vasodilation in the uterine arteries from young pregnant but not aged pregnant dams or the nonpregnant rats (Figure 2A,C). Overall, myogenic behavior was greater in the arteries of aged pregnant compared with young pregnant dams (*p* = 0.0010, Figure 2B).

### 2.3. Uterine Arteries of Aged Pregnant Dams Had Reduced Circumferential Strain and Smaller Diameters Than Uterine Arteries of Young Pregnant Dams

**Figure 3:**
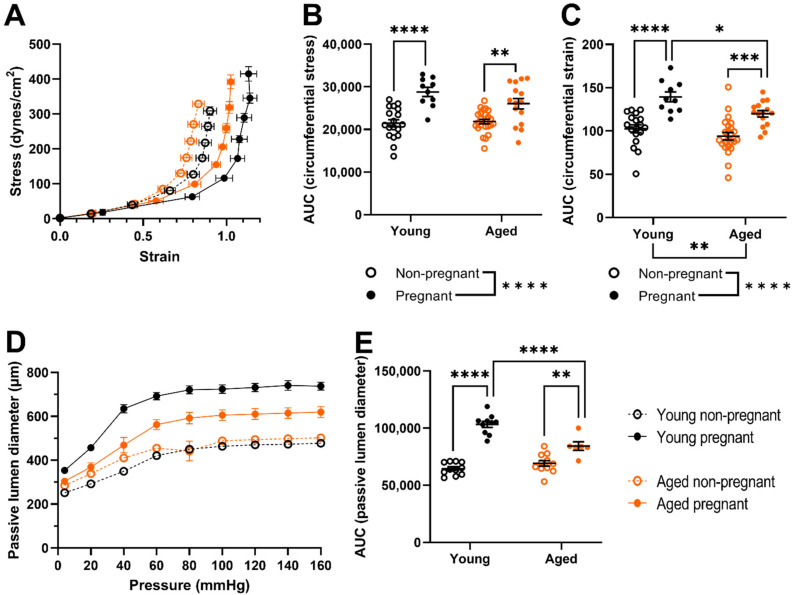
Passive (mechanical) pressure myography outcomes for main uterine arteries from young (black) and aged (orange) nonpregnant (open circles) and pregnant (GD 20; closed circles) rats. (**A**) Circumferential stress-strain; (**B**) Stress area under the curve (AUC); (**C**) Strain AUC; (**D**) Passive lumen diameter; (**E**) Passive lumen diameter AUC of arteries. All outcomes were assessed under calcium-free conditions to prevent smooth muscle cell contraction. Data presented as mean ± SEM; n = 10–24 rats/group. Outcomes were compared as AUC using a two-way ANOVA (age vs. pregnancy status) followed by Sidak’s post hoc test. * *p* < 0.05; ** *p* < 0.01; *** *p* < 0.001; **** *p* < 0.0001.

Main uterine artery circumferential stress and strain increased with pregnancy (*p* < 0.0001 overall). However, the circumferential strain of arteries from aged, pregnant dams remained lower than that of arteries from young pregnant dams (*p* = 0.0285, Figure 3A,C). There were no age-related differences in circumferential stress (Figure 3B). Passive lumen diameter increased with pregnancy in both young and aged rats (young: *p* < 0.0001, aged: *p* = 0.0011) but remained smaller in uterine arteries from aged, pregnant dams compared with young pregnant dams (*p* < 0.0001, Figure 3D,E). There was no age-related difference in arterial passive lumen diameter within the nonpregnant groups (Figure 3E).

### 2.4. Aged Rats Had a Lower Collagen:Elastin Ratio Prior to Pregnancy, Which Was Not Affected by Pregnancy

**Figure 4:**
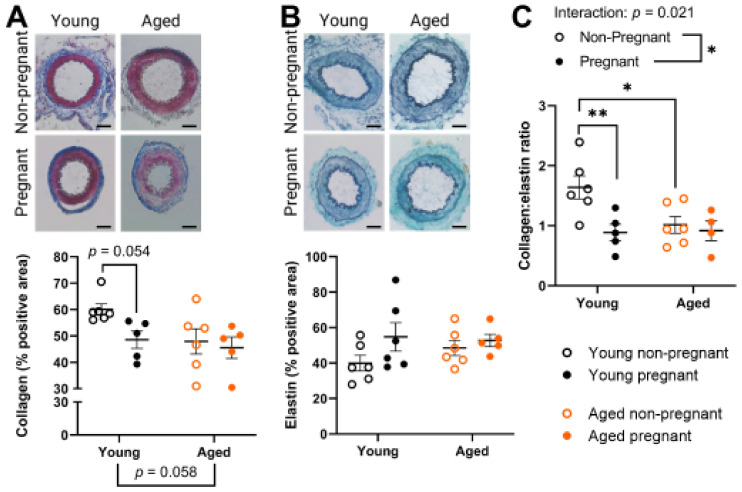
Structure of main uterine arteries from young and aged nonpregnant and pregnant (GD 20) rats. Tunica intima and media (combined) percentage positive area for (**A**) collagen (Masson’s Trichrome; collagen is stained blue) and (**B**) elastin (Verhoff stain with Fast Green counterstain; elastin fibers are stained dark), and the (**C**) collagen:elastin ratio. Scale bar represents 100 µm. Data presented as mean ± SEM; n = 4–6/group. Outcomes were compared using a two-way ANOVA (age vs. pregnancy status) followed by Sidak’s post hoc test. * *p* < 0.05; ** *p* < 0.01.

In the tunica intima and media of the main uterine artery, collagen and elastin expression were assessed due to their role in vascular structural properties and involvement in pregnancy-associated vascular remodeling [22,23,24]. Only young dams tended to reduce uterine artery collagen expression (*p* = 0.0538) due to pregnancy, whereas collagen levels tended to be reduced with age (*p* = 0.0575) (Figure 4A). The uterine artery expression of elastin did not change with age or pregnancy (Figure 4B). Overall, the collagen:elastin ratio was significantly lower in young pregnant compared to young nonpregnant rats (*p* = 0.0085, Figure 4C). In addition, uterine arteries from nonpregnant aged rats had a statistically lower collagen:elastin ratio than uterine arteries from nonpregnant young rats (*p* = 0.0207, Figure 4C). 

### 2.5. Advanced Maternal Age Did Not Detectably Alter Gelatinase Activity by MMP-2

**Figure 5:**
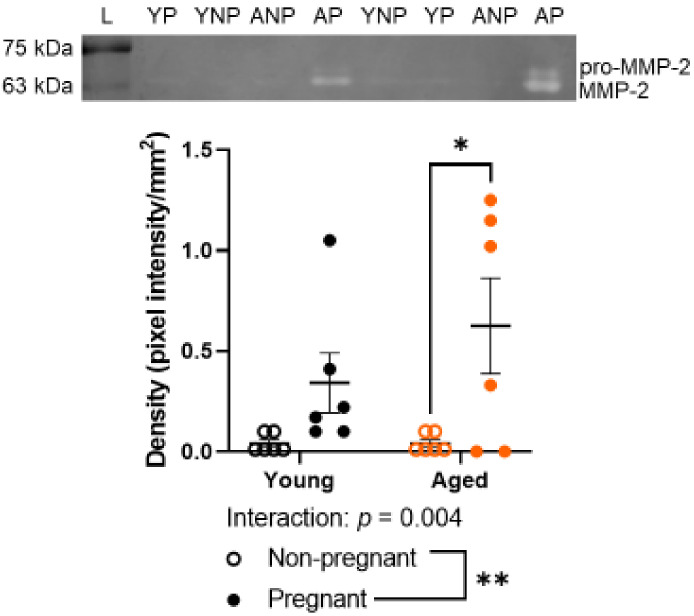
Densitometry analysis of activity by MMP-2 from main uterine arteries of young (black) and aged (orange) nonpregnant (open circles) and pregnant (GD 20; closed circles) rats. Representative zymography gel: the first lane has a protein ladder. Both pro-MMP-2 (72 kDa) and active MMP-2 (62 kDa) are visible. However, only MMP-2 bands had a sufficiently detectable band able to be used for analysis. Densitometry assessment: bands of clearing represent gelatinase activity by MMPs. L, ladder; YNP, young nonpregnant; YP, young pregnant; ANP, aged nonpregnant; AP, aged pregnant. Data presented as mean ± SEM; n = 6/group. Outcomes were compared using a two-way ANOVA (age vs. pregnancy status) followed by Sidak’s post hoc test. * *p* < 0.05; ** *p* < 0.01.

MMP-2 and MMP-9 levels were assessed due to the important role of MMP-2 and -9 in the remodeling of uterine arteries [28,39]. MMP-2 (62 kDa) activity was greater in the main uterine artery of aged pregnant dams compared to uterine arteries of aged nonpregnant rats (Figure 5, *p* = 0.0117). There was no age-related difference among nonpregnant rats. Pro-MMP-2 (72 kDa), MMP-9 (84 kDa), and pro-MMP-9 (92 kDa) were not sufficiently detectable to be analyzed within the small quantity of uterine artery protein available for zymography. Original gels are provided in Figure S1.

### 2.6. Serum Estrogen Levels Were Not Altered by Age or Pregnancy

**Figure 6:**
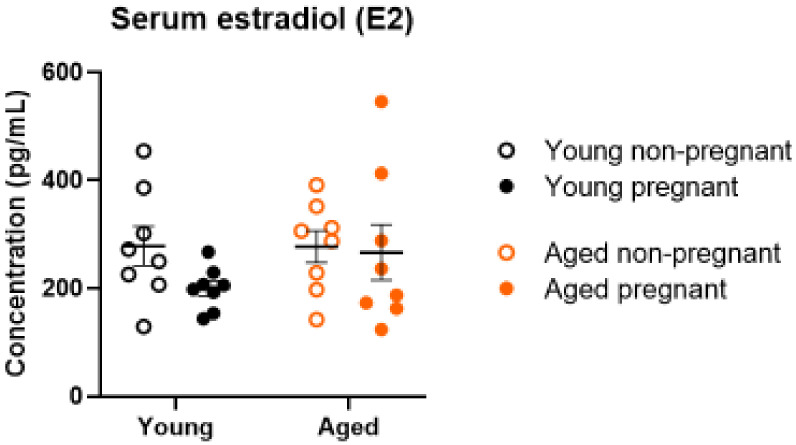
Serum estradiol (E2) concentrations of young (black) and aged (orange) nonpregnant (open circles) and pregnant (GD 20; closed circles) rats. Data presented as mean ± SEM; n = 8/group. Outcomes were compared using a two-way ANOVA (age vs. pregnancy status) followed by Sidak’s post hoc test.

Estradiol was assessed due to its role in MMP regulation [40,41,42]. Estradiol concentrations in serum did not change with pregnancy or age (Figure 6).

## 3. Discussion

In the present study, we assessed pregnancy-induced main uterine artery adaptations of function and structure in young and aged rats. Using a rat model of advanced maternal age, we showed that aging impacts both structural and functional adaptations of uterine arteries during pregnancy. Pregnancy outcomes were impaired in aged compared to young pregnancies. Forced vasodilation occurred in uterine arteries at high intraluminal pressures in only young pregnant dams and not in aged, pregnant dams. This resulted in lower myogenic behavior in the arteries of young pregnant rats compared with aged pregnant or young and aged nonpregnant rats. Circumferential strain and passive lumen diameter were lower in uterine arteries of aged pregnant than in young pregnant dams. There was a pregnancy-induced reduction in the collagen:elastin ratio of arteries from young rats, which was not observed in arteries from aged rats. MMP-2 activity was significantly increased with pregnancy in aged rats but not in the young rats. Additionally, serum estradiol levels did not change with pregnancy or age. Overall, advanced maternal age did alter several functional and structural adaptations to pregnancy that were assessed within our rat model.

Aged dams had worse pregnancy outcomes than young dams, including smaller litter sizes, more reabsorptions, and a lower fetal:placental weight ratio. These findings align with our previous observations using this rat model [38]. We now extend those findings to report that maternal age affected fetal and placental outcomes similarly to both male and female fetuses and placentas. We then assessed uterine artery function to determine whether there were any age-related changes in pregnancy adaptations that link to these poor fetal outcomes.

In the present study, at high pressures (100–160 mmHg), only the uterine arteries of young pregnant dams were overcome by excessive intravascular pressure and were no longer able to maintain vessel diameter using myogenic tone. Thus, we observed forced vasodilation in accordance with a three-phase model of arterial myogenic behavior [43]. Others have also reported forced vasodilation in young dams, such as in rat radial uterine arteries (exposed to both superfused L-NNA and indomethacin) from late pregnant dams from 140 mmHg onwards (tested up to 200 mmHg) [14]. With main uterine arteries from mice, at 110 mmHg and above, forced dilation occurred in pregnant vessels, while this did not happen in nonpregnant vessels, which maintained myogenic tone up to 160 mmHg [18]. A study of human myometrial arteries showed an increased myogenic tone of pregnant vessels until 60 mmHg, after which myogenic tone decreased slightly at higher pressures [10], indicating forced vasodilation within this group. Forced vasodilation occurs when smooth muscle cells are no longer able to generate force due to excessive intraluminal pressure [43,44,45]. The effect of pregnancy on forced vasodilation has been studied within the cerebral vasculature, in which the pressure required to elicit forced vasodilation may decrease with pregnancy [46]. To the best of our knowledge, however, the purpose of forced vasodilation within the pregnant uterine vasculature remains unknown. Occurrences such as chronic hypertension may increase the intraluminal pressure of the uterine artery, thus bringing the intraluminal pressure towards a range at which forced vasodilation may occur with an additional acute challenge. In any case, the presence of forced vasodilation in the arteries of young but not aged dams may be due to the structural differences, including a smaller luminal diameter, that we observed with aging. Thus, observing normal adaptations to pregnancy with forced dilation at higher pressures is potentially a mechanism to enhance uteroplacental blood flow, which was absent in the aged, pregnant dams.

Overall, our findings of decreased myogenic tone with pregnancy in the main uterine arteries of young rats are in line with literature from other rodent studies. For instance, pregnancy decreases the myogenic tone in main uterine arteries from guinea pigs [16] and decreases the myogenic tone of main uterine arteries in mice [17]. We have previously observed greater myogenic tone of both main uterine and mesenteric arteries from aged pregnant compared to young pregnant dams [38]. In the case of uterine arteries, this alteration of myogenic tone with advanced maternal age could affect the maintenance of constant uteroplacental blood flow with varying blood pressures.

We showed that circumferential stress (force per unit area) and strain (deformation due to stress) of main uterine arteries increased with pregnancy. The circumferential strain adaptation appeared to be less extensive in aged rats compared to young rats. This is an example of an age-related vascular difference that was only present with pregnancy. It was not seen amongst young and aged rats prior to pregnancy (between nonpregnant groups). The lower maximum uterine artery diameter of aged pregnant compared with young pregnant dams suggests that advanced maternal age results in altered vascular adaptations to pregnancy, conferring a reduced capacity for blood flow and possibly less potential to compensate for impaired vascular function when needed. We previously observed no difference in the circumferential stress and strain of mesenteric arteries in young and aged pregnant rats [38]; thus, these differences may be unique to the uterine vasculature.

We next assessed arterial collagen and elastin as they affect vascular compliance and stiffness. The observed pregnancy-related decrease in the collagen:elastin ratio within the uterine arteries of young rats in the present study appears to be due to a decreased collagen content. In line with our findings, a decrease in collagen with pregnancy has been reported in uterine arteries of (young) pigs and sheep [22,23], which facilitates luminal widening and increased compliance as blood volume greatly increases. Elastin contributes to arterial compliance by enabling greater vascular stretch (if other vascular components allow). Studies with pig, sheep, and mouse models showed no changes in elastin area with pregnancy [22,23,24,47], and degradation of elastin with age in humans only starts to occur significantly in ages 60–70 [48]. In our study, the collagen:elastin ratio decreased with pregnancy in young rats but did not decrease with pregnancy in aged rats, which demonstrates less pregnancy-induced remodeling in aged rats. This may be because prior to pregnancy, the aged rats already had a ratio similar to that of young pregnant dams. The arrangement of collagen and elastin may have also been affected by advanced maternal age, as factors such as elastin fragmentation can also increase vascular stiffness [49,50]. Overall, the existing structural differences prior to pregnancy resulted in different age-related arterial remodeling in response to pregnancy.

The degradation of elastin and collagen in the vascular wall is regulated by matrix metalloproteinases (MMPs)-2 and -9. In our study, pro-MMP-2, pro-MMP-9, and MMP-9 activity levels were below a reliable detection limit with gelatin zymography. However, we observed an increased level of MMP-2 in the main uterine arteries of pregnant dams compared to arteries of nonpregnant rats, in line with previous literature [28,39,51]. This pregnancy-related increase was greater in aged compared to young rats. Interpretation of zymography data is limited by the fact that zymography assesses the MMP gelatinase activity, not the direct activity of collagen and elastin degradation. Zymography does not assess in vivo activity but rather the maximum potential gelatinase activity in tissues as tissue inhibitors of metalloproteinases (TIMPs) and other inhibiting factors dissociate from MMPs during electrophoresis. However, our lack of finding greater levels of MMP-2 in arteries from aged rats than in arteries from young rats does not align with previous literature, as aging increases the activity of MMP-2 in the aortas of rodents, monkeys, and humans [52,53]. Indeed, MMPs and TIMPs, in general, are also known to increase with aging, which promotes degradation of the extracellular matrix [54]. This increase is long-term and likely has more profound effects, such as increased elastin degradation, contributing to the increased stiffness of vasculature with age [55]. Other pregnancy complication models, particularly reduced uteroplacental perfusion rat models, have shown reduced levels of MMP-2 and MMP-9 activity in the uterine vasculature [28,56,57]. However, it must be noted that a wide variety of MMPs exist that contribute to vascular remodeling during pregnancy, and a wide range of factors that regulate expression and activity reviewed in [58].

Due to the role of estrogen in enhancing the release of MMPs from vascular smooth muscle cells [31,35,36,37,41,42] and its negative association with advanced maternal age [32,33,34,59,60,61], we assessed serum estradiol (E2) levels in this rat model. However, we observed no significant difference in serum estradiol levels with age or pregnancy. Although estradiol increases throughout pregnancy in Sprague-Dawley rats, there is a sharp decline at the end of gestation [62], which may explain the lack of differences in estrogen levels due to pregnancy. 

Advanced maternal age affects many pregnancies and is associated with poor pregnancy outcomes. Vascular adaptations to pregnancy ensure sufficient nutrient and oxygen supply to the uteroplacental unit. Using a rat model, we have identified that advanced maternal age alters main uterine artery myogenic responses during pregnancy, such that the forced vasodilation observed in arteries of young rats was completely absent in arteries of aged rats. Additionally, circumferential strain and passive lumen diameter did not increase to the same extent with pregnancy in aged rats as in young rats. The arteries of aged rats had a lower collagen:elastin ratio prior to pregnancy, and they had a greater MMP-2 active capacity during pregnancy compared with the arteries of young rats. Thus, advanced maternal age results in functional and structural differences in one of the major arteries responsible for supplying blood to the uterus. These changes could decrease the maximum uteroplacental blood supply. We also report that some of these changes (e.g., the collagen:elastin ratio) exist before pregnancy. These age-related differences provide a potential target for future study into the prevention of poor pregnancy outcomes within this large and understudied at-risk patient population.

## 4. Materials and Methods

All experimental procedures received prior approval by the University of Alberta Health Sciences 2 Animal Care and Use Committee in accordance with the Canadian Council on Animal Care guidelines (protocols #242/2019 and #3692/2021).

### 4.1. Advanced Maternal Age Rat Model

We used a previously established rat model of advanced maternal age [38,63,64,65]. Aged rats at 9.5 months of age correspond to ~35 years of age in humans, based on milestones such as weaning, skeletal and sexual maturity, and reproductive senescence, and young control rats at 3.5–4 months of age correspond to ~early reproductive maturity in humans [63,66]. Sprague Dawley rats were purchased from Charles River at three months of age. The rats were housed in pairs within the Animal Care Facility at the University of Alberta, which maintains an ambient temperature of 22 ± 1 °C and a 14:10 h light-dark cycle. All rats were allowed at least one week to acclimatize prior to mating. After acclimatization, young rats continued to have ad libitum access to standard rat chow, while aging female rats were fed a restricted diet of six pellets per day to prevent age-related obesity from being a confounding factor. Females were randomly allocated to either nonpregnant or pregnant groups. Female rats allocated to pregnant groups (young: 3.5–4 months, aged: 8.5–9 months) were mated overnight via housing with Sprague Dawley males. Pregnancy was confirmed the next morning by the presence of a plug or sperm in a vaginal smear (designated as a gestational day (GD) 0). Dams confirmed to be pregnant by positive smear testing were single-housed and returned to an ad libitum diet.

### 4.2. Pregnancy Outcomes and Tissue Collection

Pregnant dams at GD 20 and age-matched, nonpregnant females were euthanized via exsanguination (cardiac puncture) under isoflurane-induced anesthesia. Blood was collected into heparin-coated tubes and centrifuged, then plasma was snap-frozen and stored until further use. The uterine vasculature (pregnant dams) together with the uterus (nonpregnant females only) were carefully excised and placed into ice-cold HEPES-buffered physiological saline solution (PSS; in mmol/L: 142 NaCl, 4.7 KCl, 1.17 MgSO_4_, 4.7 CaCl_2_, 1.18 K_2_PO_4_, 10 HEPES, 5.5 glucose, pH 7.4). Pregnancy outcomes (litter size, numbers of reabsorptions, placental weight, fetal sex, weight, crown-rump length, and abdominal girth; n = 13–19 dams per group) were recorded. Placental efficiency was calculated as fetal weight divided by placental weight. A small section of the isolated main uterine artery was embedded in OCT, and the remainder of the isolated main uterine artery tissue was snap-frozen. Frozen samples were stored at −80 °C until analysis.

### 4.3. Ex Vivo Assessment of Uterine Artery Vascular Function Using Pressure Myography

Functional (myogenic tone, n = 6–10 rats per group) and mechanical (circumferential stress-strain, n = 10–24 rats per group) properties were assessed using pressure myography. The right main uterine arteries were isolated from adipose tissue. A vessel segment from the mid-point of the artery was mounted on two glass cannulas in a two-bath pressure myograph (Living Systems, Burlington, NJ, US) containing PSS at 37 °C. Intravascular pressure was monitored and adjusted throughout the experiment using a pressure servo control PS/200 and a perfusion pressure monitor. Vessel and wall diameters were measured using a Lasico digital filar eyepiece (model 1602E-10) and processor attached to a stereo microscope (Olympus SZH10).

Arteries were equilibrated for 40 min, during which they were exposed to a stepwise increase in pressure from 60 to 80 mmHg, as described previously [67]. The intraluminal pressure was then adjusted to 50 mmHg for a further 10 min. Vessels were slightly pre-constricted with a low dose of phenylephrine (individually established for each vessel, mean ± SEM reduction of 21.3 ± 2.5% in lumen diameter, based on lumen diameter at 50 mmHg; mean ± SEM dose was 0.175 ± 0.045 µM; cat#P6126, Millipore Sigma) added to the tissue bath, which enabled myogenic tone development in the main uterine arteries [15]. The lumen diameter of active curves, which commenced 5 min after the addition of phenylephrine, represents the average of 2–3 measurements due to vasomotion. The intraluminal diameters (ID) of arteries were measured in 20 mmHg steps across a pressure range of 4–160 mmHg, and the vessels were left at each pressure for two minutes prior to measurement. 

Next, the arteries were fully dilated to assess passive artery characteristics, using Ca^2+^-free PSS containing EGTA (in mmol/L: 142 NaCl, 4.7 KCl, 1.17 MgSO_4_, 1.18 KH_2_PO_4_, 10 HEPES, 2 EGTA). The myograph system was flushed gently with Ca^2+^-free PSS, and the bath solution changed a minimum of four times before adding superfused papaverine (0.1 µmol/L; Sigma). Following a 20 min equilibration at 50 mmHg, passive characteristics were assessed using a pressure curve. Following a 20 min equilibration, pressure steps were repeated as described above, this time with intervals of ≥1 min between each step before measurement of the ID and wall thickness. 

Percent myogenic tone was calculated as ((ID_passive_ − ID_active_)/ID_passive_) × 100, with ID_active_ measured in PSS and ID_passive_ measured in Ca^2+^-free solution. Circumferential wall stress and strain were analyzed as described previously [38,68] and statistically compared to the area under the curve (AUC) data using GraphPad Prism (v9.0.0, San Diego, CA, US).

### 4.4. Histological Assessment of Arterial Remodeling

Main uterine artery (n = 4–6 rats per group) sections embedded in OCT were sectioned at 8 μm thickness and stained for either collagen or elastin. Collagen was visualized using Masson’s Trichrome staining on formalin-fixed sections as per kit instructions (cat#HT15-1KT, with the required additional Bouin’s solution cat#HT10132 and Weigart’s iron hematoxylin solution set cat#HT1079, Sigma Aldrich). Elastin was visualized using Verhoff staining [69]. In short, sections were first fixed in Baker’s solution (0.75 g CaCl_2_ and 7.5 mL of 37% formaldehyde with deionized water added to make a total of 250 mL) for ten minutes. Verhoff solution (0.25 g powdered hematoxylin (cat#47223, Alfa Aesar) in 5 mL of absolute ethanol, mixed well with 2 mL of 10% ferric chloride (cat#217091000, ThermoFisher Scientific) in deionized water before the addition of 2 mL of Lugol’s iodine solution (0598-05, Medisca, Richmond, BC, Canada)) was added to slides for 15 min before rinsing off with water. A 1% ferric chloride (in water) solution was added until elastin structures were clearly visible, then rinsed with water. Sections were then counterstained using Fast Green stain (cat#A16520-06, Alfa Aesar, Tweksbury, MA, USA). After staining, tissue sections were dehydrated and cover-slipped. Slides were imaged using bright field microscopy at 40× magnification (EVOS XL Core Imaging System, ThermoFisher Scientific, Waltham, MA, USA). Images were analyzed using ImageJ software (NIH), employing color deconvolution and percent positive area tools.

### 4.5. Zymography Assessment of MMP-2 and MMP-9

We performed gelatin zymography to assess the capacity for the activity of MMP-2 and -9 [41,42]. Snap-frozen isolated uterine arteries (n = 6 rats per group) were homogenized in a homogenization buffer (0.5 M Tris-HCl at pH 6.8, consisting of 1.5 M NaCl, 0.05 M CaCl_2_, 0.5% Triton-X100, 1 mM phenylmethylsulfonyl fluoride and 1% *v*/*v* protease inhibitor [100× Halt Protease Inhibitor Cocktail, cat#78429, ThermoFisher Scientific]) and sonicated. Samples were then centrifuged at 14,000 rpm for 10 min to remove cellular debris. Taking a small volume of the supernatant, the total protein of each sample was determined using the BCA protein assay, with bovine serum albumin as a standard (cat#23225, ThermoFisher Scientific, Waltham, MA, USA). The assay was read using a BioTek Synergy HTX multi-mode reader (Agilent). Each sample was mixed with 2× sample loading buffer (62.6 mmol/L Tris-HCl [pH 7.4], 25% *v*/*v* glycerol, 4% w/v sodium dodecyl sulfate, 0.01% bromophenol blue), then resolved in non-reducing 8% SDS acrylamide gel containing 0.5% gelatin (Gelatin Type A from Porcine Skin, cat#G-1890, Sigma Aldrich, Oakville, ON, Canada). 25 µg of total protein was loaded per well and separated via electrophoresis. The gels were washed 3× with 2.5% Triton-X100 for 20 min per wash to remove the SDS; then, proteins were renatured to enable the digestion of gelatin during a 19-h incubation at 37°C in a renaturing buffer (50 mM Tris, 0.5 M NaCl, 5 mM CaCl_2_, 8 mM NaN_3_). Gels were then overstained with Coomassie Brilliant Blue stain (cat#G-250, Sigma Aldrich) and destained in aqueous 25% methanol and 10% acetic acid. Gels were imaged using an Odyssey Classic and analyzed using Odyssey V3.0 software (Li-cor Biosciences, Lincoln, NE, USA).

### 4.6. Assessment of Serum Estradiol Levels

The concentration of estradiol (E2) in serum (diluted 1:1 in PBS; n = 8 rats per group) was measured using an ELISA kit (cat#EKN45082, BioMatik), as per the manufacturer’s guidelines. Samples were added in duplicates, and the plate was read with a BioTek Synergy HTX multi-mode reader (Agilent). Data were analyzed using a 4-parameter logistic curve. The sample optical density values used to calculate the estradiol concentration from the standard curve were first corrected by subtracting the optical density of the blank wells. Values were then corrected for the dilution, and duplicates were averaged.

### 4.7. Statistical Analysis

Data were analyzed using GraphPad Prism (v9.0.0, San Diego, CA, USA). The effect of age on reabsorptions was assessed using a Mann-Whitney U test, and the effect on other pregnancy outcomes was assessed using a *t*-test. For all vascular outcomes, the effects of age and pregnancy status were assessed using a two-way ANOVA with Sidak’s post-hoc test. Grubb’s test was used to exclude significant outliers in the data sets. Data are presented as mean ± SEM; *p* < 0.05 was considered statistically significant. For the pregnancy outcomes, n represents the number of dams.

## Data Availability

Data is contained within the article or Supplementary Material.

## Supplementary Materials

The supporting information can be downloaded at: https://www.mdpi.com/article/10.3390/ijms23169191/s1.
