# Supplementary material for: Advanced Maternal Age Impairs Uterine Artery Adaptations to Pregnancy in Rats
Source: Int J Mol Sci. 2022 Aug 16;23(16):9191. doi: 10.3390/ijms23169191 (PMC9409016; doi:10.3390/ijms23169191)
Supplement: Supplementary file 1 [file ijms-23-09191-s001.zip › ijms-1854507-supplementary.pdf]

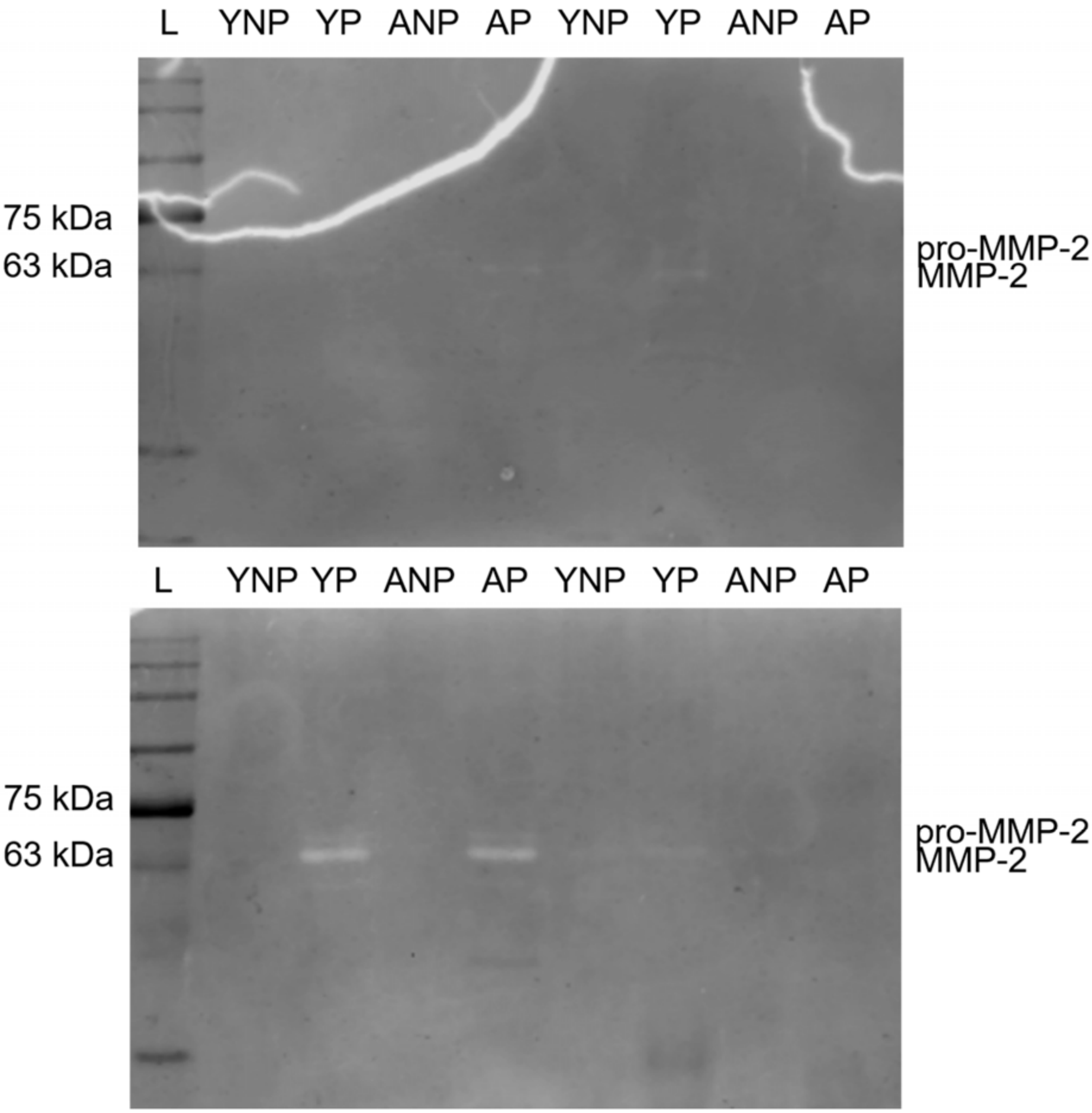
L
YNP
YP
ANP
AP
YNP
YP
ANP
AP
75 kDa
63 kDa
pro-MMP-2
MMP-2
L
YNP
YP
ANP
AP
YNP
YP
ANP
AP
75 kDa
63 kDa
pro-MMP-2
MMP-2

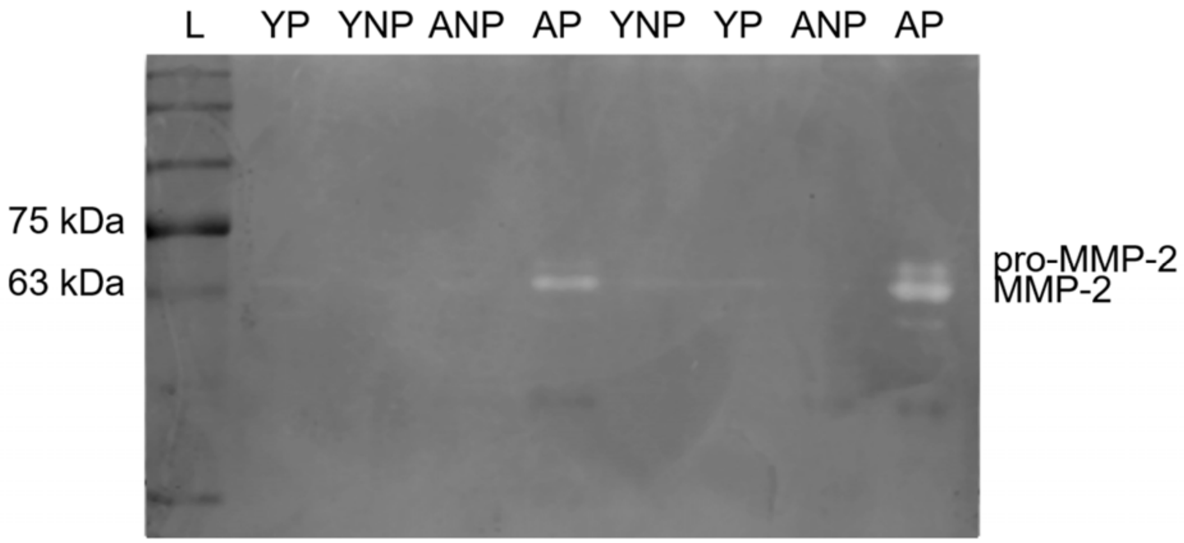

Figure S1. Original images for densitometry analysis of activity by MMP-2 from main uterine arteries of young and aged non-pregnant and pregnant (GD 20) rats. Zymography gels: the first lane has a protein ladder. Both pro-MMP-2 (72 kDa) and active MMP-2 (62 kDa) are visible, however, only MMP-2 bands had a sufficiently detectable band able to be used for analysis. Densitometry assessment: bands of clearing represent gelatinase activity by MMPs. L, ladder; YNP, young non-pregnant; YP, young pregnant; ANP, aged non-pregnant; AP, aged pregnant.
